# Unraveling the mysteries of serum albumin—more than just a serum protein

**DOI:** 10.3389/fphys.2014.00299

**Published:** 2014-08-12

**Authors:** Angelica M. Merlot, Danuta S. Kalinowski, Des R. Richardson

**Affiliations:** Molecular Pharmacology and Pathology Program, Department of Pathology, Faculty of Medicine, Bosch Institute, The University of SydneySydney, NSW, Australia

**Keywords:** serum albumin, albumin-binding proteins, albumin receptors, gp60, tumors, albumin drug carriers

## Abstract

Serum albumin is a multi-functional protein that is able to bind and transport numerous endogenous and exogenous compounds. The development of albumin drug carriers is gaining increasing importance in the targeted delivery of cancer therapy, particularly as a result of the market approval of the paclitaxel-loaded albumin nanoparticle, Abraxane®. Considering this, there is renewed interest in isolating and characterizing albumin-binding proteins or receptors on the plasma membrane that are responsible for albumin uptake. Initially, the cellular uptake and intracellular localization of albumin was unknown due to the large confinement of the protein within the vascular and interstitial compartment of the body. Studies have since assessed the intracellular localization of albumin in order to understand the mechanisms and pathways responsible for its uptake, distribution and catabolism in multiple tissues, and this is reviewed herein.

## Serum albumin

### Structure

**Figure 1:**
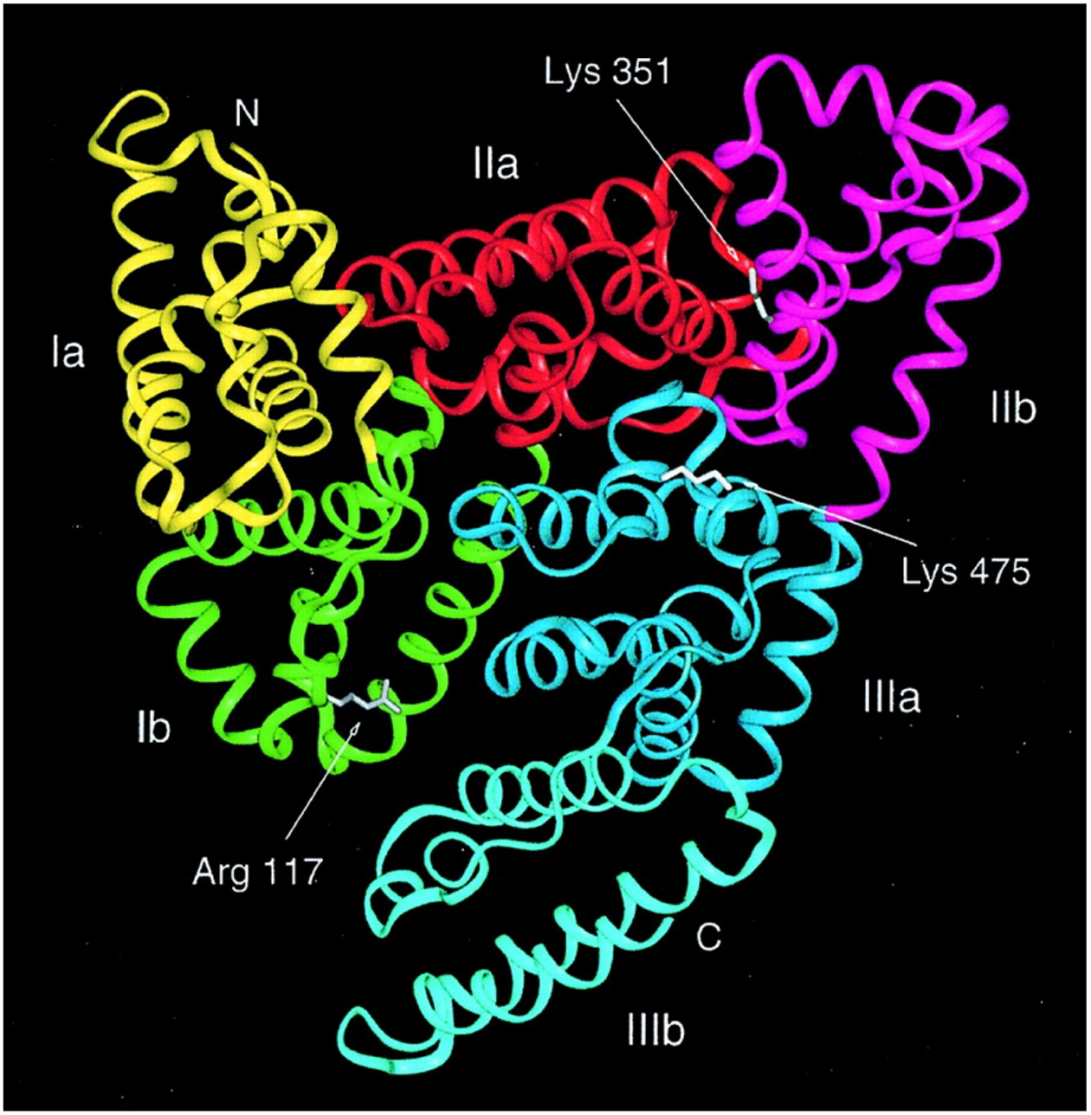
**Structure of human serum albumin consisting of three domains, each grouped into subdomains A and B (Subdomain Ia, yellow; Ib, green; IIa, red; IIb, magenta; IIIa, blue; and IIIb, cyan)**. Sugio et al. (1999) by permission of Oxford University Press.

Serum albumin is the most abundant protein in the blood plasma of all vertebrates with the concentration in human serum being 35–50 mg/mL (Peters, 1996). Human serum albumin (HSA) has a molecular mass of 66,348 Da and is composed of three homologous domains, numbered I, II, and III (Figure 1) (He and Carter, 1992; Peters, 1996; Sugio et al., 1999). Each domain is grouped into subdomains A and B that possess common structural motifs. The two principal regions responsible for ligand-binding to HSA are known as Sudlow's Site I and II, located in subdomain IIA and IIIA (Figure 1), respectively (Sudlow et al., 1976; Peters, 1996). Albumin is coded by a single gene, which is expressed in a co-dominant manner with both alleles being transcribed and translated (Hawkins and Dugaiczyk, 1982; Peters, 1996). The human albumin gene is located on the long arm of chromosome 4 at position q13.3.

### Function

Albumin is primarily synthesized by the liver with the human liver producing ~13.9 g of HSA per day (Peters, 1996). HSA has an approximate half-life of 19 days and is degraded more effectively if it is denatured or structurally altered (Peters, 1996). Albumin has a variety of important functions and is responsible for 80% of the colloidal osmotic pressure of blood (Peters, 1996). Significantly, albumin is able to bind various endogenous molecules, including long-chain fatty acids, steroids, L-tryptophan, etc. (Kragh-Hansen, 1981; Peters, 1996; Evans, 2002). Moreover, albumin is also involved in transporting ions in the circulation, including copper, zinc, calcium, etc. (Peters, 1996).

Additionally, this vital protein is able to bind exogenous compounds and drugs, such as warfarin, ibuprofen, chlorpromazine and naproxen, with the affinity of their binding significantly affecting their activity and half-life (Kragh-Hansen, 1981; Peters, 1996; Evans, 2002). Furthermore, albumin also acts as a toxic waste carrier, binding bilirubin, the product of heme breakdown, to deliver it to the liver for hepatic excretion (Peters, 1996). Interestingly, albumin is also believed to act as an anti-oxidant on account of its ability to: (1) protect bound substances from peroxidative damage (e.g., fatty acids and lipoproteins); and (2) bind free copper, limiting its redox activity and the production of free radicals (Peters, 1996; Evans, 2002). Lastly, albumin is a source of thiols that are avid reactive oxygen and nitrogen species scavengers (Peters, 1996; Evans, 2002).

### Distribution

Interestingly, albumin is predominately present in the extravascular space (~242 g) rather than the intravascular space (~118 g) (Peters, 1996; Evans, 2002). In fact, the protein is prevalent in extracellular locations such as skin, gut, muscle, other fluids (i.e., cerebrospinal, pleural, etc.) and secretions (e.g., sweat, tears and milk) (Peters, 1996). However, very low concentrations of albumin are present intracellularly (Peters, 1996). Albumin returns from the extravascular space to the circulation *via* the lymphatic system, making ~28 “trips” in and out of the lymphatic system during its lifetime (Peters, 1996; Evans, 2002).

Upon secretion from hepatocytes, albumin enters the circulation and translocates to the extracellular space through the pores of sinusoidal or fenestrated endothelium in certain organs, such as the liver, pancreas, small intestine and bone marrow (Peters, 1996). However, in organs where a continuous endothelium predominates, it is now believed that albumin can traverse the endothelium *via* active transcytotic mechanisms, including receptor-mediated mechanisms (e.g., albondin; see Section entitled “Cellular Albumin-Binding Proteins”).

## Accumulation of albumin in the tumor interstitium

**Figure 2:**
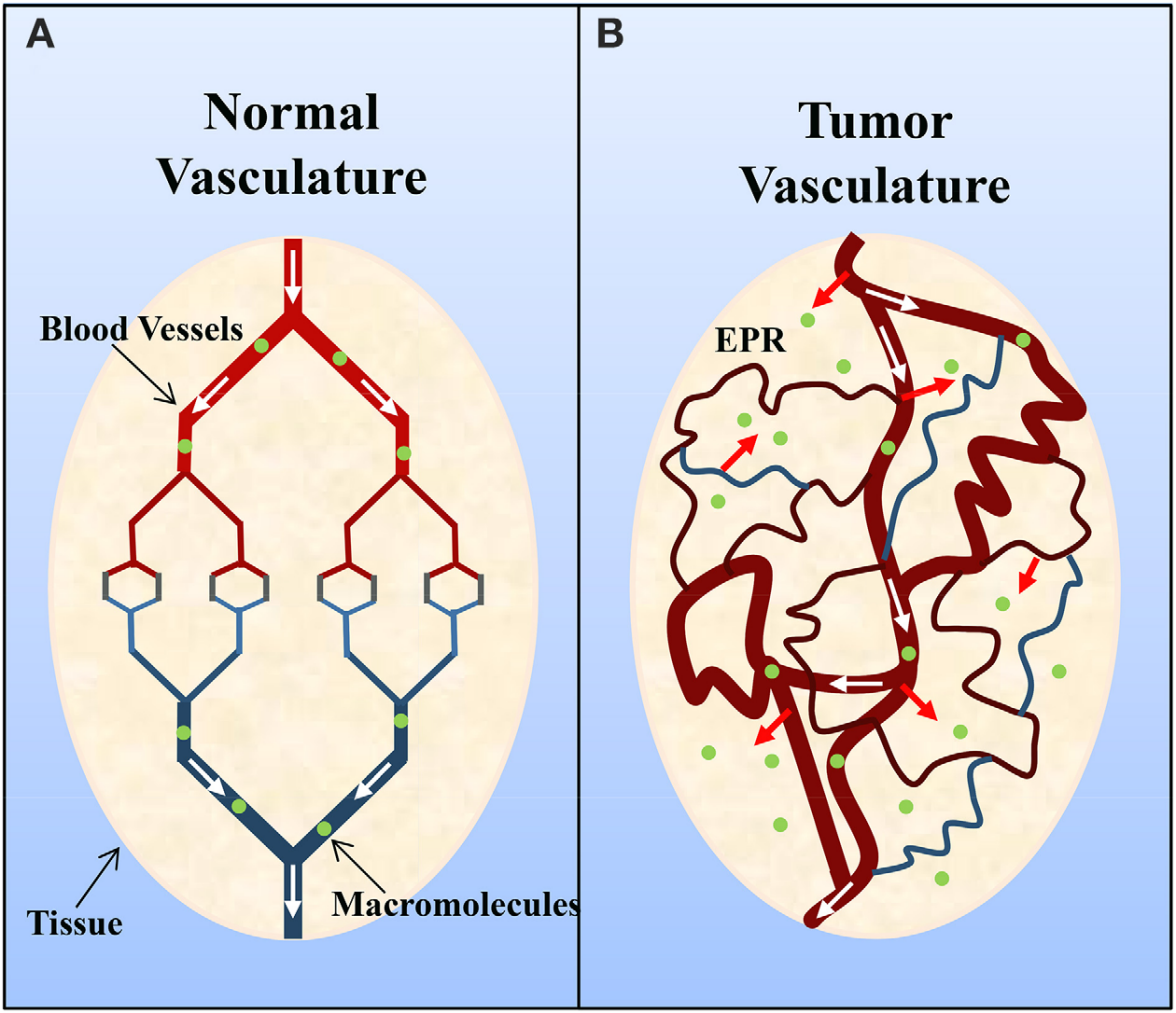
**Schematic representation of (A) normal and (B) tumor vasculature**. Normal tissue is composed of mature, organized blood vessels, while tumor tissue consists of immature, leaky and tortuous vessels. The altered organization of tumor vasculature and disorganized lymphatic network results in vascular leakage and the accumulation of macromolecules (>40 kDa) within the interstitium and is known as the enhanced permeation and retention (EPR) effect. Adapted by permission from Macmillan Publishers Ltd: Nature Medicine (Jain, 2001), copyright (2001).

Solid tumors commonly possess an immature, highly permeable vasculature that is acted upon by vascular permeability-enhancing factors (e.g., nitric oxide) (Carmeliet and Jain, 2000; Maeda et al., 2000; Greish, 2007; van der Veldt et al., 2008). However, despite this there is generally insufficient lymphatic drainage (Carmeliet and Jain, 2000; Maeda et al., 2000; Greish, 2007). This subsequently results in an accumulation of macromolecules (>40 kDa) within the tumor interstitium, and this is known as the enhanced permeation and retention effect (Figure 2) (Maeda et al., 2000; Greish, 2007). Of interest, Matsumura and Maeda (1986) demonstrated that an intravenously injected Evans blue-albumin complex accumulated in sarcoma 180 tumors of ddY mice. The retention of albumin in tumors has since been observed in various experimental solid tumors (e.g., sarcoma, ovarian carcinoma, Novikof hepatoma, etc.) using radiolabeled- or dye-complexed serum albumin (Peterson and Appelgren, 1973; Sinn et al., 1990; Andersson et al., 1991; Schilling et al., 1992; Stehle et al., 1997; Wunder et al., 1997).

Additionally, a number of studies have proposed that tumors are a site of albumin catabolism (Hradec, 1958; Andersson et al., 1991; Schilling et al., 1992; Stehle et al., 1997). For instance, in a mouse sarcoma model (C57/RL6J) injected with ^3^H-raffinose-labeled albumin, at least 2–3-fold greater levels of ^3^H were observed in the lysosomes of tumors when compared with lysosomes of normal tissue (Andersson et al., 1991). Furthermore studies have demonstrated that albumin has a shorter half-life and a higher turnover in tumor-bearing mice, despite a compensatory increase in hepatic albumin synthesis, compared to non-tumor-bearing mice (Hradec, 1958). Hence, it has been suggested that tumors utilize albumin as a source of energy, by breaking down albumin into its component amino acids in lysosomes that are subsequently used by cancer cells for their accelerated growth (Stehle et al., 1997). Moreover, studies have suggested that the hypoalbuminiemia evident in cancer patients is a result of albumin catabolism by the tumor (Stehle et al., 1997).

Nevertheless, some of these earlier studies suffer from several experimental limitations. For instance, it is difficult to obtain pure lysosomal fractions and, thus, it is necessary to reproduce these studies and test the purity of fractions using well established membrane and organelle markers (Graham, 2002; Yamagishi et al., 2013). Additionally, several other *in vivo* factors may affect albumin degradation and catabolism (e.g., levels of corticosteroids) (Peters, 1996). Therefore, a clear-cut relationship has not been established and additional *in vivo* studies are necessary to support the intracellular distribution and catabolism of albumin by tumors.

More recently, Commisso et al. observed that cancer cells harboring endogenous oncogenic Ras mutations have increased levels of macropinocytosis *in vitro* and *in vivo* (Commisso et al., 2013). Moreover, it was demonstrated that FITC-labeled albumin was internalized through macropinocytosis and subsequently resulted in increased levels of glutamate and α-ketoglutarate in oncogenic Ras-transformed cells (Commisso et al., 2013). Interestingly, the decrease in proliferation of oncogenic Ras-expressing cells after glutamine deprivation was shown to be rescued by extracellular albumin supplementation (Commisso et al., 2013). These findings suggest that macropinocytosis of albumin provides nutrients to sustain cancer cell proliferation (Commisso et al., 2013).

## Cellular albumin-binding proteins

Considering the importance of albumin, a number of putative albumin-binding proteins and receptors have been identified in various tissues and cell lines (Table 1), including kidney (Zhai et al., 2000; Amsellem et al., 2010), endothelium (Schnitzer and Bravo, 1993), fibroblasts (Porter et al., 1995), and tumor-cell surfaces (Fritzsche et al., 2004). Specifically, seven membrane-associated albumin-binding proteins have been discovered, namely: albondin/glycoprotein 60 (gp60) (Schnitzer et al., 1988), glycoprotein 18 (gp18) (Ghinea et al., 1988), glycoprotein 30 (gp30) (Ghinea et al., 1988), the neonatal Fc receptor (FcRn) (Roopenian and Akilesh, 2007), heterogeneous nuclear ribonucleoproteins (hnRNPs) (Fritzsche et al., 2004), calreticulin (Fritzsche et al., 2004), cubilin (Zhai et al., 2000; Amsellem et al., 2010), and megalin (Zhai et al., 2000; Amsellem et al., 2010). Moreover, a secreted albumin-binding protein known as secreted protein, acidic and rich in cysteine (SPARC) has been identified (Schnitzer and Oh, 1992). Considering their importance in albumin uptake by cells, each of these proteins are described in detail below.

**Table 1:** **Localization of albumin-binding proteins and receptors**.

**Protein/Receptor**	**Tissue**	**Substrate**
Albondin/gp60	Continuous endothelium	Native albumin
gp18	Endothelium, macrophages, fibroblasts and MDA-MB-453 breast cancer cell surfaces	Modified-albumin
gp30	Endothelium, macrophages, fibroblasts and MDA-MB-453 breast cancer cell surfaces	Modified-albumin
SPARC	Endothelial cells, vascular smooth muscle cells, skeletal muscle, fibroblasts, testicular, ovarian, pancreatic and a range of tumor cells	Native albumin
hnRNPs	Human tumor cell lines: CEM T-cell leukemia cells, MCF-7 breast cancer cells and MV3 melanoma cells	Native albumin
Calreticulin	Human tumor cell lines: CEM T-cell leukemia cells, MCF-7 breast cancer cells and MV3 melanoma cells	Native albumin
FcRn	Endothelium, antigen-presenting cells, gut, kidneys, lungs and the blood-brain-barrier (central nervous system endothelium and choroid plexus)	Native albumin
Cubilin	Kidney proximal tubule cells, absorptive intestinal cells, placenta, and visceral yolk-sac cells	Native albumin and probably modified-albumin
Megalin	Kidney proximal tubule cells, absorptive intestinal cells, placenta, visceral yolk-sac cells, choroid plexus, thyrocytes, ciliary epithelium, lungs, parathyroid, endometrium, oviduct, inner ear, and epididymal epithelial cells	Native albumin and probably modified-albumin

### Albondin/gp60

Albondin (gp60) is a 60 kDa glycoprotein that acts as an albumin receptor that is widely distributed, but is selectively expressed on the plasma membrane of continuous endothelium (except for the brain), where it operates to increase capillary permeability (Ghinea et al., 1988, 1989; Schnitzer et al., 1988; Schnitzer, 1992; Schnitzer and Oh, 1994; Tiruppathi et al., 1996). Albondin not only specifically binds native albumin, but also facilitates its internalization and subsequent transcytosis (Milici et al., 1987; Schnitzer, 1992; Schnitzer and Oh, 1994; Tiruppathi et al., 1996).

It has been proposed that ~50% of albumin leaves the capillary lumen *via* albondin, with the remainder traversing this barrier through intercellular junctions and/or fluid-phase mechanisms (Schnitzer, 1993; Schnitzer and Oh, 1994). Moreover, it has been demonstrated that the internalization of albondin occurs through a caveolin-dependent endocytotic process that results in transcytosis and does not appear to enter the degradative endosome-lysosome system (Schnitzer, 1993; Schnitzer and Bravo, 1993; Schnitzer et al., 1995; Tiruppathi et al., 1997; Iancu et al., 2011).

### gp18 and gp30

Both gp18 and gp30 avidly bind conformationally-modified albumin (i.e., gold-labeled albumin, formaldehyde- or maleic-anhydride-treated albumin) and do not preferentially interact with native albumin, similarly to other known scavenger receptors (Ghinea et al., 1989; Schnitzer and Oh, 1992, 1994; Schnitzer et al., 1992; Schnitzer and Bravo, 1993). Unlike albondin, gp18 and gp30 are found on a variety of cells, such as macrophages and fibroblasts, and a range of endothelia (Schnitzer et al., 1992). Moreover, gp18 has been observed to be expressed in human MDA-MB-453 breast cancer cells (Wang et al., 1994). These scavenger receptors bind and direct modified albumins for degradation, perhaps as part of protein catabolism or as a protective pathway to remove altered, old, damaged or potentially deleterious albumins (Schnitzer, 1993; Schnitzer and Bravo, 1993). Albumin may be modified through oxidation, non-enzymatic glycation, maleylation, etc. as a result of normal aging or as a protective or pathological response (Schnitzer, 1993; Peters, 1996). Denatured or modified albumin is degraded faster and more efficiently than native albumin, suggesting that these alterations select albumin molecules for degradation (Peters, 1996).

### SPARC

SPARC is also known as osteonectin and BM-40 and is secreted by several cell types (Brekken and Sage, 2001). Interestingly, SPARC has been found to be highly expressed in malignant cells and stromal cells associated with neoplasia (Porter et al., 1995; Podhajcer et al., 2008). SPARC possesses albumin-binding properties and specifically interacts with native albumin in a similar way to albondin, but differing from 18 to gp30 that bind conformationally altered albumin (Schnitzer and Oh, 1992). Specifically, anti-SPARC antibodies also recognize albondin, but not 18 or gp30, suggesting that SPARC and albondin share a native albumin-binding domain (Schnitzer and Oh, 1992). However, there is no evidence that SPARC mediates albumin uptake into tumors. It has been postulated that the ability of SPARC to bind albumin in the tumor interstitium enhances the accumulation of albumin-bound drugs within the tumor space (Desai et al., 2008, 2009). Moreover, a preliminary clinical trial demonstrated that SPARC expression correlated with the response to paclitaxel-loaded albumin nanoparticle (*nab*-paclitaxel or Abraxane®) treatment, with SPARC-positive patients having a better clinical outcome (Desai et al., 2009). However, conflicting data in a KPfC mouse model has challenged this hypothesis, as SPARC deficiency did not alter the intra-tumoral concentrations of Abraxane® (Neesse et al., 2014). Consequently, further studies are necessary to validate this hypothesis, including larger clinical trials involving a greater number of patients. Currently, a phase III study (NCT00785291), by the National Cancer Institute, is evaluating whether SPARC expression in serum predicts patient response to Abraxane®, and this may further our understanding of the role of SPARC in albumin accumulation by tumors.

### hnRNPs and calreticulin

Five different albumin-binding proteins have been identified from plasma membranes of human cancer cells lines (i.e., CCRF-CEM T-cell leukemia, MV3 melanoma and MCF7 breast carcinoma) (Fritzsche et al., 2004). Four of these proteins were identified as members of the hnRNP family, including hnRNP A2/B1, hnRNP C1, hnRNP A1 and hnRNP A3, and the fifth protein was found to be calreticulin (Fritzsche et al., 2004). Calreticulin was first described as an endoplasmic reticulum chaperone and calcium signaling protein, but has since been shown to be involved in several cellular functions, including cell adhesion, modulation of platelet-collagen interactions (wound healing) and apoptosis (Mendlovic and Conconi, 2010). The functions of the hnRNP family are not well characterized. However, most members of the hnRNP family have been described as nuclear RNA-binding proteins involved in pre-mRNA processing, such as RNA splicing, export and stability (Chaudhury et al., 2010). Interestingly, hnRNPs have been proposed to play a role in carcinogenesis where their over-expression acts as biomarkers for the early detection of tumors (Han et al., 2013). The significance of these findings is currently unclear and it remains to be determined whether these proteins are involved in albumin-mediated uptake.

### FcRn

FcRn is expressed in multiple cell-types and tissues, including antigen-presenting cells, vascular endothelium, gut, lungs, kidneys and the blood-brain barrier (BBB) (i.e., endothelium and choroid plexus) (Roopenian and Akilesh, 2007). This receptor protects albumin and IgG, from degradation by binding both proteins with high affinity only at a low pH (pH < 6.5) in acidic endosomes, preventing their degradation *via* the lysosomal pathway and returning them to the extracellular space (pH 7.4) (Chaudhury et al., 2003; Ober et al., 2004; Anderson et al., 2006; Andersen et al., 2012). This consequently extends the half-life of serum albumin (Chaudhury et al., 2003; Anderson et al., 2006; Sarav et al., 2009). The role of this receptor in albumin uptake by tumors remains unclear.

### Cubilin and megalin

Cubilin is a multi-ligand receptor that is most recognized for its involvement in the intestinal uptake of the intrinsic factor vitamin B_12_ complex (Seetharam et al., 1997). Moreover, cubilin has been shown to be involved in the endocytosis and transcellular transport of numerous ligands, including albumin (Birn et al., 2000). Cubilin is localized to absorptive intestinal cells, placenta, visceral yolk-sac cells and proximal tubules of kidneys (Christensen and Birn, 2002). Megalin is a large trans-membrane protein that has also been shown to bind albumin (Cui et al., 1996). This protein is more widely expressed than cubilin, being present in the choroid plexus, kidney proximal tubule cells, thyrocytes, etc. (Table 1) (Christensen and Birn, 2002).

Interestingly, megalin binds to cubilin with high affinity and it has been suggested that megalin contributes to the internalization of cubilin-ligand complexes as a co-receptor (Moestrup et al., 1998; Christensen and Birn, 2002). Moreover, cubilin also binds amnionless, a protein that is necessary for the expression of cubilin on the cell membrane (Amsellem et al., 2010). Cubilin, in conjunction with megalin, has an essential role in the uptake of albumin (i.e., reabsorption) by the proximal tubules of the kidneys (Zhai et al., 2000; Amsellem et al., 2010). Cubilin- and/or megalin-deficiency in mice and dogs was shown to cause a decrease in the uptake of albumin in the proximal tubule resulting in albuminuria (Birn et al., 2000; Amsellem et al., 2010). Additionally, patients with Imerslund-Gräsbeck syndrome, caused by a mutation in the cubilin gene, in general suffer from proteinurea, demonstrating the importance of cubilin in protein renal reabsorption (Grasbeck, 2006).

## Albumin as a drug carrier in oncology

Considering the enhanced permeation and retention effect and the accumulation of albumin in the tumor interstitium, the development of albumin as a drug carrier is increasingly important to consider in terms of the targeted delivery of cancer therapy (Kratz, 2008, 2010). It has also been proposed that albumin carriers take advantage of the presence of albondin on the endothelium and SPARC in the tumor interstitium to increase the accumulation of drugs in the tumor space (Desai et al., 2009; Kratz, 2010). Various drug delivery systems with albumin have been developed including albumin nanoparticles, drug albumin conjugates, albumin-binding drug derivatives and prodrugs (for reviews see Kratz, 2008, 2010).

The development and market approval of the paclitaxel-loaded albumin nanoparticle, *nab*-paclitaxel or Abraxane®, was a major breakthrough in the field of albumin carrier development. Abraxane® was initially approved for clinical use in the United States in 2005 (Kudlowitz and Muggia, 2014). This albumin nanoparticle is indicated for the treatment of metastatic breast cancer, after failure of combination chemotherapy (Kudlowitz and Muggia, 2014). More recently, Abraxane® has also been described for the first-line treatment of patients with metastatic adenocarcinoma of the pancreas, in combination with gemcitabine, and patients with locally advanced or metastatic non-small cell lung carcinoma, in combination with carboplatin (Kudlowitz and Muggia, 2014). Abraxane® has a greater therapeutic index than paclitaxel alone, being administered at higher doses with less toxicity and more efficacy than traditional paclitaxel therapy (Gradishar et al., 2005; Socinski et al., 2012; Iwamoto, 2013). Abraxane® is currently still being further evaluated in clinical trials for other tumors, such as cancer of the bladder (NCT00583349) and multiple myeloma (NCT02075021).

Moreover, albumin-binding as a general strategy for improving the pharmacokinetics of drugs is also being assessed. Traditionally, the binding of a drug to albumin is believed to reduce the level of free drug available to exert its therapeutic activity (Lancon et al., 2004; Vuignier et al., 2010). However, studies have also demonstrated mechanisms by which albumin acts to effectively improve therapeutic use or reduce rapid clearance (Dennis et al., 2002; Merlot and Richardson, 2014). For instance, the experimental anti-cancer thiosemicarbazone, namely di-2-pyridylketone 4,4-dimethyl-3-thiosemicarbazone (Dp44mT) (Merlot et al., 2013a), has been shown to be internalized by cancer cells *via* a putative carrier/receptor (Merlot et al., 2013b; Merlot and Richardson, 2014). Interestingly, the uptake, toxicity and apoptotic activity of Dp44mT is greatly enhanced in the presence of HSA (Merlot and Richardson, 2014). Considering Dp44mT targets lysosomes to induce apoptosis (Lovejoy et al., 2011), and that HSA potentially undergoes lysosomal catabolism in tumors (Andersson et al., 1991; Stehle et al., 1997), it can be hypothesized that HSA facilitates Dp44mT delivery to the lysosomes, enhancing its anti-cancer activity (Merlot and Richardson, 2014). Although studies are yet to identify the exact mechanism of the HSA-stimulated uptake process, albumin-binding may provide an advantage when generating tumor targeting agents and this requires further intense investigation.

## Conclusion

Albumin is a versatile and captivating protein. In view of the large confinement of albumin within the vascular and interstitial space, the intracellular distribution of albumin has remained poorly characterized for many years. It may be possible that albumin, under specific conditions or during cellular stress, is taken up by normal cells and tumor cells at low and high levels, respectively, due to their metabolic rate. The exact role of some incompletely characterized albumin-binding proteins (i.e., hnRNPs and calreticulin) in mediating albumin uptake remains to be determined. However, the search and characterization of albumin-binding proteins, particularly in cancer cells, is of considerable interest in light of the development of albumin as an effective drug carrier to target tumors.

### Conflict of interest statement

The authors declare that the research was conducted in the absence of any commercial or financial relationships that could be construed as a potential conflict of interest.
